# Functional organization and restoration of the brain motor-execution network after stroke and rehabilitation

**DOI:** 10.3389/fnhum.2015.00173

**Published:** 2015-03-30

**Authors:** Sahil Bajaj, Andrew J. Butler, Daniel Drake, Mukesh Dhamala

**Affiliations:** ^1^Department of Physics and Astronomy, Georgia State UniversityAtlanta, GA, USA; ^2^Department of Physical Therapy, Byrdine F. Lewis School of Nursing and Health Professions, Georgia State UniversityAtlanta, GA, USA; ^3^Department of Veteran's Affairs, Atlanta Rehabilitation Research and Development Center of ExcellenceDecatur, GA, USA; ^4^Neuroscience Institute, Joint Center for Advanced Brain Imaging, Center for Behavioral Neuroscience, Georgia State UniversityAtlanta, GA, USA

**Keywords:** functional magnetic resonance imaging, spectral Granger causality, brain network activity, stroke recovery, low-frequency oscillations

## Abstract

Multiple cortical areas of the human brain motor system interact coherently in the low frequency range (<0.1 Hz), even in the absence of explicit tasks. Following stroke, cortical interactions are functionally disturbed. How these interactions are affected and how the functional organization is regained from rehabilitative treatments as people begin to recover motor behaviors has not been systematically studied. We recorded the intrinsic functional magnetic resonance imaging (fMRI) signals from 30 participants: 17 young healthy controls and 13 aged stroke survivors. Stroke participants underwent mental practice (MP) or both mental practice and physical therapy (MP+PT) within 14–51 days following stroke. We investigated the network activity of five core areas in the motor-execution network, consisting of the left primary motor area (LM1), the right primary motor area (RM1), the left pre-motor cortex (LPMC), the right pre-motor cortex (RPMC) and the supplementary motor area (SMA). We discovered that (i) the network activity dominated in the frequency range 0.06–0.08 Hz for all the regions, and for both able-bodied and stroke participants (ii) the causal information flow between the regions: LM1 and SMA, RPMC and SMA, RPMC and LM1, SMA and RM1, SMA and LPMC, was reduced significantly for stroke survivors (iii) the flow did not increase significantly after MP alone and (iv) the flow among the regions during MP+PT increased significantly. We also found that sensation and motor scores were significantly higher and correlated with directed functional connectivity measures when the stroke-survivors underwent MP+PT but not MP alone. The findings provide evidence that a combination of mental practice and physical therapy can be an effective means of treatment for stroke survivors to recover or regain the strength of motor behaviors, and that the spectra of causal information flow can be used as a reliable biomarker for evaluating rehabilitation in stroke survivors.

## Introduction

Hemiparesis is one of the most common deficits observed following stroke (Dromerick and Reding, 1995). The functional imbalance within the motor system following stroke (James et al., 2009; Wang et al., 2010; Grefkes and Fink, 2011; Inman et al., 2012) can be due to damage of the white axonal tracts connecting brain motor areas (Turken et al., 2008; Granziera et al., 2012). Due to limited clinical data compared to healthy volunteers data, recovery and restorative brain mechanisms in stroke survivors (SS) are not clearly understood. Specifically, scientists have yet to identify specific node and network activities of damaged brains that are invoked and/or restored following rehabilitative treatments. Mental practice (MP) and physical therapy (PT) are two evidence-based interventions currently used to improve motor movement, coordination and balance following stroke (Page et al., 2002; Jackson et al., 2004; Butler and Page, 2006). MP or motor imagery (MI) represents mental rehearsal of a motor action without any overt action; and has been shown to improve motor behaviors in people with neurologic disorders (Sharma et al., 2006, 2009). Likewise various forms of PT have been shown to be effective in ameliorating motor weakness following stroke (Wolf et al., 2006; Langhorne et al., 2011). Here, we studied the brain network mechanism for motor function recovery as a result of: MP only, and MP and PT in combination.

Low frequency oscillations (LFOs) (<0.1 Hz) in blood-oxygenation-level dependent (BOLD) functional magnetic resonance imaging (fMRI) signals reflect self-organizing dynamic behavior of the brain. Several cortical and subcortical regions, including motor regions M1, PMC, and SMA, interact and coordinate within and across the hemispheres within the low frequency (<0.1 Hz) range during resting-state (Wu et al., 2011; Bajaj et al., 2014). The origin and functional relevance of these oscillations have not been completely investigated (Cordes et al., 2001; Buzsáki and Draguhn, 2004; De Luca et al., 2006; Razavi et al., 2008). An emerging, well-accepted notion is that these slow intrinsic fluctuations are believed to be associated with neural level excitability changes in cortical and subcortical networks (Buzsáki and Draguhn, 2004; Balduzzi et al., 2008; Keilholz et al., 2010) which provides neural substrates for the flexibility and variability in cognition and motor behaviors (Arieli et al., 1996; Palva and Palva, 2012). These slow coherent oscillations are the backbone of whole-brain functional connectivity networks such as default-mode networks (Raichle et al., 2001; Buckner et al., 2008), which are actively being investigated in basic and clinical neuroscience (Fox and Greicius, 2010; Gillebert and Mantini, 2013). Despite tremendous progress in revealing these network patterns in resting-state and clinical cases, the spectral features of oscillatory network activity and their modulations in patients by task conditions or therapy are not completely understood.

Recent neuroimaging studies (Cordes et al., 2000; Solodkin et al., 2004; Grefkes et al., 2008a; Kasess et al., 2008) have extensively studied the brain motor networks during resting-state (RS), motor imagery (MI) and motor execution (ME) and have shown that overlapping networks are engaged in these task conditions. Planning, initiation, guidance and coordination of voluntary movements could modulate functional connectivity in the motor networks in these tasks (Jiang et al., 2004). The motor network commonly includes these areas: the primary motor area (M1), the premotor cortex (PMC) and the supplementary motor area (SMA) (Jeannerod and Frak, 1999; Gerardin et al., 2000; Kasess et al., 2008), which taken together play a dominant role in the development, specification and execution of action. Activity in these cortical areas during resting-state is thought to maintain a dynamic equilibrium but is modulated during a motor task by disturbing the balance and coordination of cortical areas by inhibiting each other (Jiang et al., 2004). The primary motor area (M1) is one of the principle brain areas that generates and sends neuronal signals to control the execution of motor commands whereas secondary motor areas SMA and PMC are involved in motor planning, sending neuronal impulses to M1. Also, the functional and potential asymmetries in PMC play an important role in controlling interhemispheric interactions during bimanual motor task (Berg et al., 2010). Anatomically, M1 is connected to SMA and PMC in the same as well as in the opposite hemisphere allowing bilateral activity during rest, unimanual and bimanual hand movements (Schell and Strick, 1984; Deecke, 1987; Rouiller et al., 1994; Bajaj et al., 2014).

In this study, we used the spectral version of Granger causality technique (Geweke, 1982; Dhamala et al., 2008a,b) to investigate how the oscillatory network activity in the low frequency band (<0.1 Hz) within the motor network reorganizes in aged stroke survivors compared to young able-bodied participants as these stroke survivors undergo two interventions, mental practice and combined mental practice and physical therapy. Granger causality and its spectral version have been in continuous use because it is a data-driven approach where causal interactions are inferred directly from simultaneously recorded physiological data and has the capability to infer the relation among structural connectivity, functional connectivity and behavior (Seth, 2005; Seth and Edelman, 2007). The motor network we studied included: the left M1 (LM1), the right M1 (RM1), the left PMC (LPMC), the right PMC (RPMC) and the SMA. We predict that the interventions to improve motor performance in stroke survivors would change the characteristic features of the brain motor network activity in such a way as to have network commonalities with those of able-bodied healthy participants. The strength of oscillatory network activity would correlate with improvement in motor behaviors independent of intervention or in either intervention. We tested this hypothesis by examining and comparing the brain motor network activity in people recovering from stroke following interventions and healthy controls from intrinsic BOLD fMRI measurements.

## Materials and methods

### Participants

We recorded resting-state fMRI data from a total of 30 adult participants: 17 young able-bodied (all right-handed, 12 males, mean age 25.17 ± 4.68 years) and 13 aged stroke survivors (12 right-handed, 9 males, mean age 59.23 ± 9.49 years). A written consent was obtained from each participant before the experiment. The experimental protocol had appropriate institutional review boards (IRB) approval.

#### Able-bodied participants

All the participants had no abnormal neurological history. None of them reported use of medication known to affect any neurological function.

#### Stroke survivors

To be included in the study, all stroke survivors had to be at least 18 years old, independent in standing, toilet transfer, and the ability to maintain balance for at least 2 min with arm support. Upper extremity movement criteria included the ability to actively extend the affected wrist ≥20° and extend 2 fingers and thumb at least 10° with a motor activity log (MAL) score of less than 2.5 (Uswatte et al., 2006). All of them survived their first stroke within 54 months prior to enrollment. Either MR imaging or computed tomography (CT) was used to confirm stroke and its location (Table 1). Stroke latency ranged from 1 to 54 months. Six of them had left hemiparesis resulting from infarct or hemorrhage located in the thalamus, basal ganglia, internal capsule, caudate, and/or pre-central gyrus. The remaining volunteers had right hemiparesis due to infarctions of the middle cerebral, pontine or internal carotid arteries (Table 1) (Inman et al., 2012). The Mini-Mental State Exam (MMSE) was used to assess cognitive aspects of mental function where a maximum score of 30 describes normal cognition function (Folstein et al., 1975) (Table 1). This measure constituted two sets of questions; one set tested orientation, memory and attention whereas the second set tested the participant's ability to name, follow verbal and written commands, write a sentence spontaneously and copy a complex polygon. The Fugl–Meyer Motor Assessments (FMA) was used to assess sensation and motor functions. This included a total of 33 items including reflexes, volitional movement assessment, flexor synergy, extension synergy, movement combining synergies, movement out of synergy, normal reflex assessment, wrist movement, hand movement, co-ordination and speed, each with a scale from 0 to 2 (0 for no performance, 1 for partial performance and 2 for complete performance) (Fugl-Meyer et al., 1975). The total possible score was 66 where a score of nearly 33 represents moderate impairment of the affected upper limb.

**Table 1:** **Clinical and demographic data of the stroke group**.

**Participant**	**Age (years)**	**Sex**	**Post stroke (months)**	**MMSE**	**Stroke location**
1	55	F	5	30	L thal. hem.
2	55	M	1	27	L basal ganglia
3	52	M	8	24	R cingulate gyrus infarct
4	74	F	9	30	R caudate infarct
5	65	F	7	28	L caudate infarct
6	54	M	11	27	R putamen hem.
7	50	M	5	30	R lacunar infarct (Globus pallidus)
8	69	F	8	28	R motor cortex infarct
9	64	M	54	28	R basal ganglia, thalamic hem.
10	42	M	5	30	R pontine infarct
11	55	M	7	28	L internal capsule
12	62	M	7	28	L thalamic hem.
13	73	M	5	28	L pontomedullary

*M, male; F, female; MMSE, Mini-Mental State Exam*.

### Intervention details

Six participants were randomized to “mentally practice” a series of upper limb functional motor tasks for 4 h per day (8–30 min sessions), with the guidance of an audio tape, for a total of 60 h over 3 weeks. MP is the creation by the mind referring to an experience, which can be auditory, visual, tactile or kinesthetic representing movement without any physical movement. Seven participants were randomized to undergo physical training + MP. The PT + MP group underwent 15 days (4 h per day) of intensive one-on-one therapy, consisting of listening to the same MP tape for 60 min per day plus 3 h of physical therapy per day. Identical tapes were given to all participants and the six mental practice tasks did not change, but small details of the mental practice scenarios such as the type of drink or color/type of telephone one reached for were altered to enhance motivation and lessen boredom.

The MP consisted of imagining four basic MI tasks using the affected or unaffected hand. For instance, participants were asked: (1) to imagine brushing or combing their hair, (2) to imagine picking up and bringing different types of fruit to their mouth, (3) to imagine extending their arm to pick up a cup from a cabinet and place it on the counter and gently release it, and (4) to imagine cleaning the kitchen counter using a cloth.

The PT consisted of repetitive, task-oriented training of the more-impaired upper extremity for several hours a day (depending on the severity of the initial deficit). Task-oriented training involved functionally based activities performed continuously for a period of 15–20 min (e.g., writing in a journal). In successive periods of task training, the spatial requirement of the activity, or other parameters (such as duration), were changed to require more demanding control of limb segments for task completion. Feedback about overall performance was provided at the end of the 15–20 min period. A large bank of tasks was created for use among participants. Frequent rest intervals were provided through the training session.

All sessions had identical contact durations and were monitored by a licensed rehabilitation specialist. The investigators were blind to group assignment. Following the 3-week “training” period all participants underwent a second testing session recording both clinical and physiologic measures.

### Imaging

All the participants were instructed to keep their eyes open fixated on a cross in the center of a screen, relax and try not to fall asleep. Each of the able-bodied participants underwent one resting-state fMRI (rs-fMRI) scanning session. Imaging was performed using a 3-Tesla Siemens whole-body MRI scanner. Functional imaging was 7 min and 54 s long, and included a T2^*-weighted^ echo planner imaging (EPI) sequence [echo time (TE) = 40 ms; repetition time (TR) = 2000 ms; flip angle = 90°; field of view (FOV) = 24 cm, matrix = 64 × 64; number of slices = 33 and slice thickness = 5 mm]. High-resolution T1-weighted images were acquired for anatomical references using an MPRAGE sequence with an isotropic voxel size of 2 mm. Stroke participants underwent two rs-fMRI scanning sessions. The second session was executed following an intervention where stroke participants underwent either mental practice (MP) alone or mental practice combined with physical therapy (MP+PT). The gap between the sessions ranged from 14 to 51 days. Their fMRI data was collected from a Siemens 3.0 T Magnetom Trio scanner (Siemens Medical Solutions, USA) and included TR/TE/FA = 2350 ms/28 ms/90°, 130 time points (~5 min each), resolution = 3 × 3 × 3 mm^3^ and 35 axial slices.

### Data analysis

#### FMRI preprocessing

FMRI data were preprocessed by using SPM8 (Wellcome Trust Centre for Neuroimaging, London; http://www.fil.ion.ucl.ac.uk/spm/software/spm8/). The preprocessing steps involved slice time correction, realignment, normalization and smoothing. Motion correction to the first functional scan was performed within participant using a six-parameter rigid-body transformation. Six motion parameters (three translational and three rotational) were stored and used as nuisance covariates. Four able-bodied participants out of total 17 had either more than 2 mm of translation or more than 1.5° of rotation about the three axes and were excluded from the analysis. The mean of the motion-corrected images was then co-registered to the individual structural image using a 12-parameter affine transformation. The images were then spatially normalized to the Montreal Neurological Institute (MNI) template (Talairach and Tournoux, 1988) by applying a 12-parameter affine transformation, followed by a nonlinear warping using basis functions (Ashburner and Friston, 1999). Images were subsequently smoothed with an 8-mm isotropic Gaussian kernel and band-pass-filtered (0.04–0.1 Hz) in the temporal domain.

#### Regions of interest

Regions of interest (ROIs) for motor-execution network were defined using seed-based correlation mapping procedure to assess functional connectivity among the regions. The left primary motor area (LM1) was selected as seed region with a 6 mm radius sphere centered at (−33.0, −19.8, 52.1) in the MNI coordinate system. Voxel-wise BOLD time-series for all the regions were extracted by making masks using MARSBAR (http://marsbar.sourceforge.net/). The correlated regions to the LM1 were right primary motor cortex (RM1) centered at (35.7, −18.1, 52.0), left pre-motor cortex (LPMC) centered at (−34.3, −1.4, 55.8), right pre-motor cortex (RPMC) centered at (35.1, 0.1, 54.9) and midline supplementary motor area (SMA) centered at (0.0, −4.2, 64.7). Co-ordinates chosen were in accordance with one of the previous studies (Inman et al., 2012). Previously, power spectra for data with TR>2 s showed peak at frequency less than 0.04 Hz due to motion parameters (Razavi et al., 2008). Therefore, in current analysis, we extracted data from all the regions, linearly detrended and band-pass filtered within the frequency range of 0.04–0.1 Hz.

#### Spectral Granger causality measures

Spectral Granger causality measures, one of the directed functional connectivity measures (Friston et al., 2012), are a subset of spectral interdependency measures (Dhamala, 2014). Spectral interdependency measures are used to quantify the inter-relationship between oscillatory processes as a function of frequency of oscillations. It consists of three sub-measures: total interdependence (M_1,2_) (say between oscillatory processes 1 and 2), one-way directional influence either from 1 to 2 (M_1→2_) or 2 to 1 (M_2→1_) and instantaneous causal flow (M_1.2_) (Granger causality measures), which are derived from a spectral density matrix (S) and are related by equation:

(1) $$M_{1,2}=M_{1\to 2}+M_{2\to 1}+M_{1.2}$$

These are well-accepted measures to characterize frequency specific interdependence between multiple neurophysiological time-series data.

Spectral matrix (S) is constructed parametrically from the time-series of oscillatory systems using autoregressive (AR) modeling (Dhamala et al., 2008b). Diagonal elements of the matrix, S represent node activity in terms of spectral power as a function of frequency whereas directional influences i.e., Granger causality (GC) between 1 and 2 are given by:

(2) $$\begin{array}{l} M_{1\to 2}\left(f\right)\;=\;\ln\frac{S_{22}\left(f\right)}{{\tilde{H}}_{11}\left(f\right)\Sigma_{11}{\tilde{H}}_{11}^{*}\left(f\right)}\;\\ M_{2\to 1}\left(f\right)\;=\ln\frac{S_{11}\left(f\right)}{{\tilde{H}}_{22}\left(f\right)\Sigma_{22}{\tilde{H}}_{22}^{*}(f)} \end{array}$$

where ${\tilde{H}}_{11}=H_{11}+\frac{\Sigma_{12}}{\Sigma_{11}}H_{12},{\tilde{H}}_{22}=H_{22}+\frac{\Sigma_{12}}{\Sigma_{22}}H_{21}$ represent new transfer function matrices for 1 and 2 respectively in terms of noise covariance matrix, Σ and transfer function matrix, H. These are estimated from the residual errors and the inverse of the Fourier transforms of the coefficients in autoregressive models respectively.

#### Significant tests and percentage difference

*GC*-values were integrated over the frequency range from 0.04 Hz (f) to 0.1 Hz (f):

(3) $$iGC_{1\to 2}=\frac{1}{f_{2}-f_{1}}\int_{f_{1}}^{f_{2}}M_{1\to 2}\left(f\right)df$$

Thresholds for significance level of Granger causality for each participant—able-bodied (AB), stroke survivors (SS), stroke survivors under treatments: mental practice (MP) and mental practice and physical therapy (MP+PT) were computed by random permutation method (Hayasaka and Nichols, 2004). We considered AB condition as reference level for SS to calculate percentage difference (D) in connectivity strength. SS was used as reference for MP and MP+PT to calculate percent modulation (M) after treatment of MP and MP+PT. This percent difference (D) and percent modulation (M) for SS and for MP and MP+PT respectively were calculated as follows (Bajaj et al., 2014):

(4) $$D=\frac{iGC_{SS}-iGC_{AB}}{iGC_{AB}}\times 100\%$$

(5) $$M=\frac{iGC_{MP/MPPT}-iGC_{SS}}{iGC_{MP/MPPT}}\times 100\%$$

Here *iGC_SS_*, *iGC_AB_*, *iGC_MP_*, and *iGC_MPPT_* represent integrated causal flow for stroke survivors (SS) (no treatment), able-bodied (AB) participants, stroke survivors with treatment of mental practice (MP) only and stroke survivors with combined treatment of mental practice and physical therapy (MP+PT) respectively.

## Results

### Power and GC spectra

**Figure 1:**
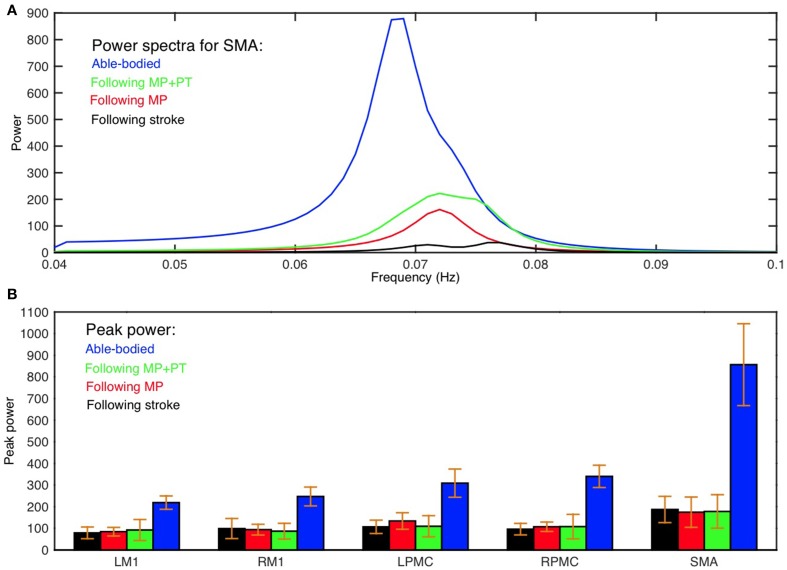
**Power spectra and peak power. (A)** Peak of power spectra for SMA occurs within the frequency band 0.06–0.08 Hz for able-bodied participants (blue colored plot), stroke survivors who underwent MP+PT (green colored plot), stroke survivors who underwent MP only (red colored plot) and for stroke survivors before intervention (black colored plot). **(B)** Peak power and the associated standard error of the mean for each ROI in each condition is shown.

**Figure 2:**
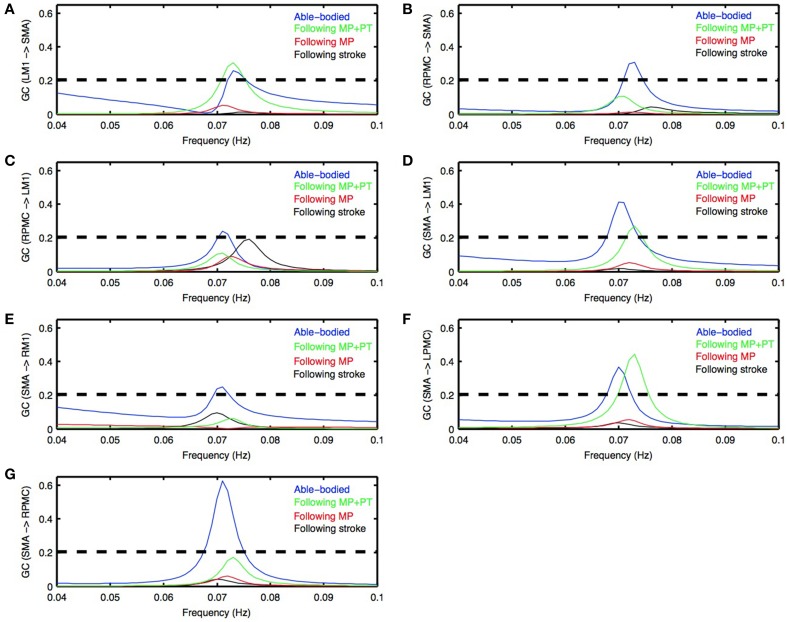
**Granger causality (GC) spectra**. Young able bodied vs. aged stroke survivors before and after intervention. Average GC spectra for all the possible connections among five ROIs (LM1, RM1, LPMC, RPMC, and SMA) were computed. Seven connections **(A–G)** were found which were significantly stronger for AB condition (blue colored plots) whereas none of the connections was significantly stronger for stroke survivors following stroke (black colored plots) as well as following MP (red colored plots). Here black colored dashed horizontal line represents the significance level (*p* < 0.01, sample size = 26) calculated using random permutation test. Three connections **(A,D,F)** were significantly stronger for participants who underwent MP+PT (green colored plots). Peak of GC spectra for all the ROIs under all the conditions was also found in the frequency range 0.06–0.08 Hz.

Average power spectra from all subjects for all five ROIs (LM1, RM1, LPMC, RPMC, and SMA) and average GC spectra from all subjects for each connection were computed for AB, SS, MP, and MP+PT conditions. Figure 1A shows group level comparison of power spectra of SMA for AB, SS, MP, and MP+PT conditions and for other ROIs, see supplementary section. In all four conditions, for all the ROIs, the peaks for power were in the frequency band 0.06–0.08 Hz. Figure 1B shows a comparison of peak power of all ROIs for all conditions with standard error of mean. The peaks for GC spectra were also found in the same frequency band 0.06–0.08 Hz (Figure 2).

### Directed functional connectivity

**Figure 3:**
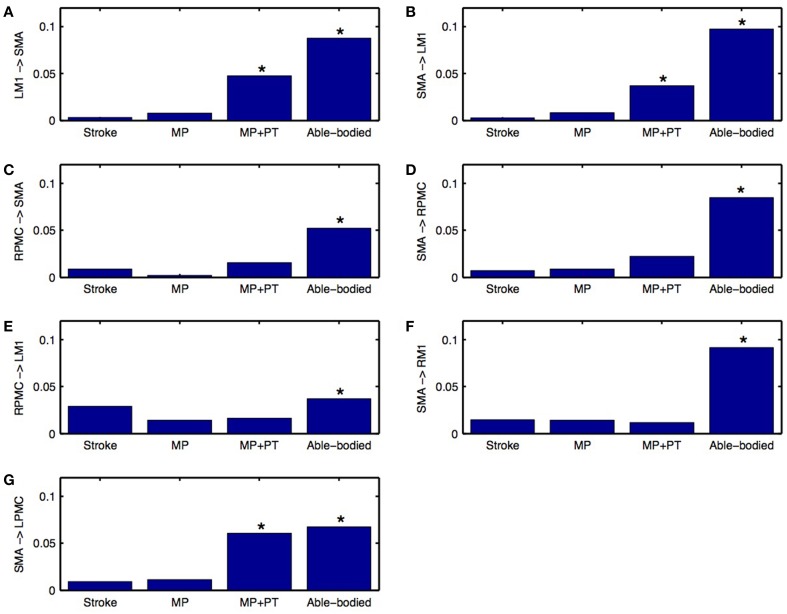
**Integrated causal flow**. Young able bodied vs. aged stroke survivors before and after intervention. Integrated causal flow for frequency band 0.04–0.1 Hz is calculated for all the seven connections **(B–G)**. Here ^*^represents significant causal flow values. Three connections: **(A)** LM1 to SMA, **(B)** SMA to LM1, and **(G)** SMA to LPMC showed significant causal flow values for stroke survivors after MP+PT whereas none of the causal influences for stroke survivors are significant before and after MP treatments.

Directed functional connectivity among five ROIs was computed for AB, SS, MP, and MP+PT conditions. For AB, seven connections were found that had significant causal flow (Figures 2A–G), including bidirectional causal flow between LM1 and SMA (Figures 2A,D; blue line) and between RPMC and SMA (Figures 2B,G; blue line). Here dashed line shows a significant threshold (*p* < 0.01, sample size = 26) calculated from combined set of data for AB and SS using random permutation test. Other connections having significant causal flows were from RPMC to LM1 (Figure 2C; blue line), SMA to RM1 (Figure 2E; blue line) and SMA to LPMC (Figure 2F; blue line). Compared to AB, the stroke survivors did not show significant causal flow (Figures 2A–G; black lines). Compared to AB, stroke survivors who underwent MP only did not demonstrate any connections with significant causal flow (Figures 2A–G, red line). On the other hand, stroke survivors who underwent combined MP+PT showed three connections, bidirectional connection between LM1 and SMA (Figures 2A,D; green line) and from SMA to LPMC (Figure 2F; green line), with significant causal flows. Integrated causal flow for all seven connections was calculated by using Equation (3) (Figures 3A–G). Significant causal flows are marked with ^*^p < 0.01, sample size = 26.

### Connectivity modulations

**Figure 4:**
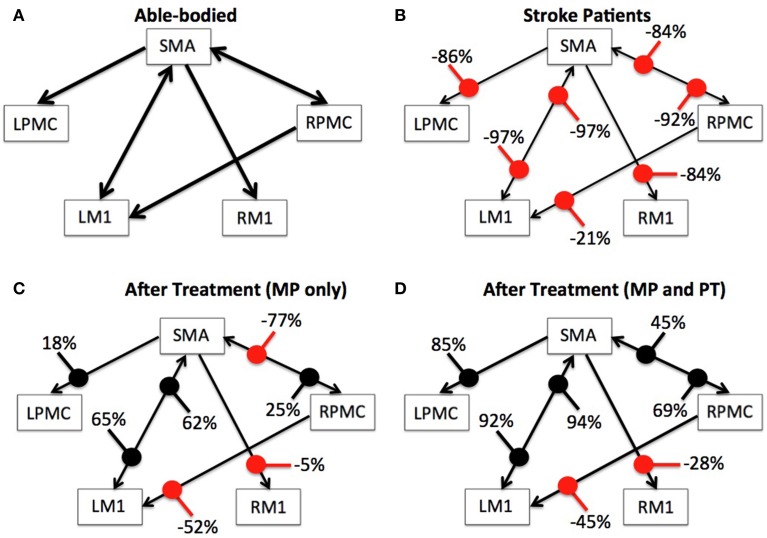
**Percent difference and modulation**. Compared to **(A)** able-bodied participants, percent decrease of the causal flow ranged from −21 to −97% for aged stroke patients as shown in **(B)**, whereas compared to these stroke patients, there was a percent modulation ranging from −77 to 65% for stroke patients who underwent MP as shown in **(C)** and from −45 to 94% for stroke patients who underwent MP+PT as shown in **(D)**. Percent decrease and percent modulations are shown with red and black colored dots respectively. Width of the arrows represents corresponding strength of connections on an arbitrary scale. Wider the arrows, stronger are the connections.

We used Equation (4) to compute percent difference (D) in connection strength for aged stroke-survivors (SS) with respect to young able-bodied (AB) people. We found that the strength of all the connections, which showed significant causal flow in AB, decreased and ranged from −21 to −97% (Figures 4A,B). Connection between SMA and LM1 was the most negatively affected connection for aged stroke-survivors. We used Equation (5) to compute the percent modulation (M) of stroke survivors, who had either MP or MP+PT treatment. We found that percent modulation for MP ranged from 18 to 65% (Figure 4C). The most affected connection found previously (between LM1 and SMA) was modulated by 62–65%. Three connections, from SMA to RM1, RPMC to LM1, and RPMC to SMA were negatively modulated by 5, 52, and 77%, respectively. We found that percent modulation for MP+PT ranged from 45 to 94% (Figure 4D). Here the most affective connections were modulated by 92–94%, which is much higher than during MP only. Two connections, from SMA to RM1 and RPMC to LM1 were negatively modulated by 28 and 45%, respectively. Percent decrease and percent modulations in Figures 4B–D are shown with red and black colored dots, respectively.

**Figure 5:**
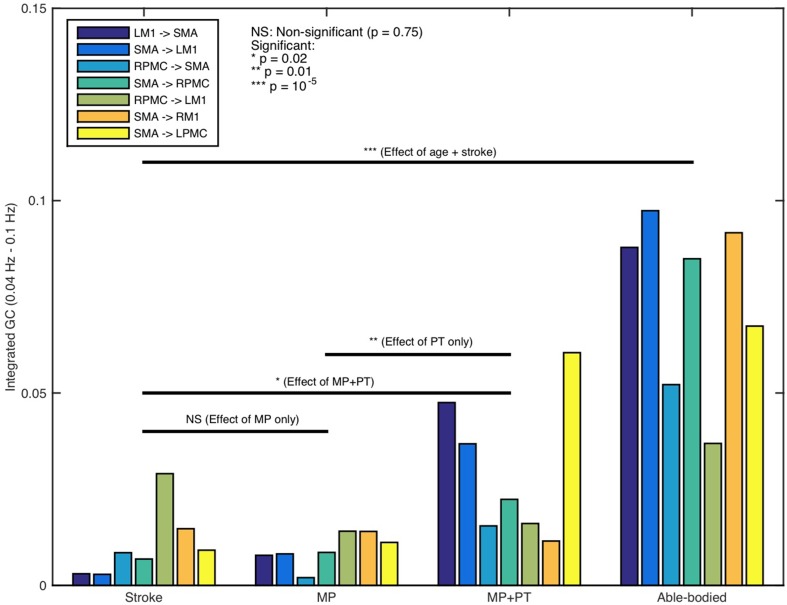
**Network activity comparison**. Considering the causal influences for all significant connections, stronger network activity (***p = 10^−5^) was observed for able-bodied participants than stroke-survivors. No significant difference between integrated causal flow values was found between stroke survivors before and after mental practice (MP) (*p* = 0.75) whereas network activity was significantly higher when they underwent combined session of mental practice and physical therapy (MP+PT) (*p = 0.02). We also found that the network activity was significantly higher following MP+PT than following MP only (**p = 0.01).

We were also interested in whether or not the behavior of the network differs for AB, SS, MP, and MP+PT groups and therefore we combined all seven individual significant connections as part of one network and performed two-sample (un-paired) *t*-test for AB vs. SS, SS vs. MP, SS vs. MP+PT, and MP vs. MP+PT (Figure 5). We found that the network as a whole, consisting of seven significant connections, was significantly stronger for young able-bodied volunteers than for aged stroke-survivors (*p* = 10^−5^, sample size = 13, denoted by ^***^). We also found that there was no significant difference between the strength of networks when the stroke survivors had only performed MP (*p* = 0.75, denoted by NS) whereas the network became significantly stronger when the stroke survivors underwent combined, MP+PT (*p* = 0.02, denoted by ^*^). We also found that the effect of MP+PT was significantly stronger than MP only (*p* = 0.01, denoted by ^**^).

### Brain and behavior correlation

**Figure 6:**
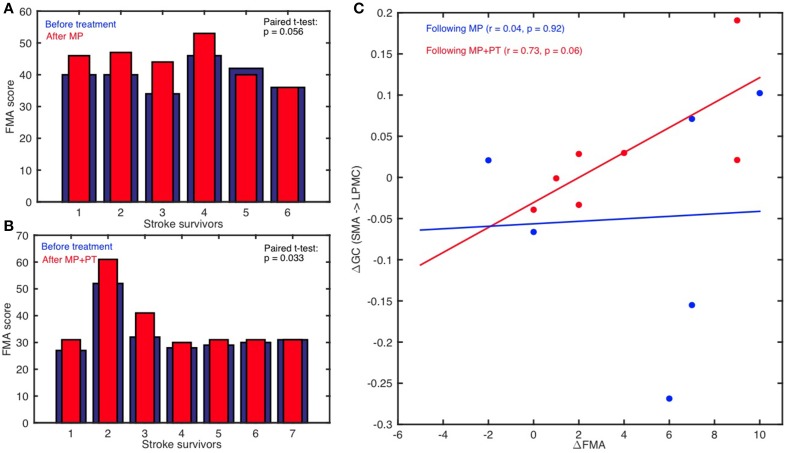
**Brain and behavioral correlation**. The Fugl-Meyer Motor Assessment (FMA) scores for stroke-survivors: **(A)** before intervention (blue bars) and after MP (red bars), and **(B)** before intervention (blue bars) and after MP+PT (red bars) are plotted. We also observed that for connection: **(C)** from SMA to LPMC, the correlation coefficient (r) between differences in FMA scores (ΔFMA) and *GC*-values (ΔGC) before and after MP+PT intervention showed a trend toward statistical significance.

FMA scores were recorded for all the stroke-survivors before and after the intervention. Using paired *t*-test, we found that FMA scores were not significantly higher when the participants underwent MP only (sample size = 6; *p* >0.05) (Figure 6A) whereas scores were significant higher when the participants underwent MP+PT (sample size = 7; *p* < 0.05) (Figure 6B). For the brain and behavior correlation, behavioral FMA score differences (ΔFMA) and brain GC differences (ΔGC) (Table 2) were not significantly correlated for the causal influence from SMA to LPMC (Figure 6C) in case of MP treatment, but tended toward significant values (*p* = 0.06, Figure 6C) for this connection in case of MP+PT treatment.

**Table 2:** **Differences in FMA scores (ΔFMA) and GC values (ΔGC) before and after the intervention**.

**Participant**	**ΔFMA**	**ΔGC (following intervention-following stroke)**		
		**LM1 → SMA**	**SMA → LM1**	**SMA → LPMC**
** PARTICIPANTS WHO UNDERWENT MP ONLY: MP-STROKE**				
1	6	−0.36	−0.23	−0.26
2	7	0.00	0.08	0.07
3	10	−0.06	0.02	0.10
4	7	−0.01	−0.12	−0.15
5	−2	−0.01	0.00	0.02
6	0	0.01	−0.03	−0.06
** PARTICIPANTS WHO UNDERWENT MP+PT: (MP+PT)-STROKE**				
7	4	0.04	0.00	0.02
8	9	0.1	0.10	0.02
9	9	−0.02	0.04	0.19
10	2	0.00	−0.17	0.02
11	2	0.09	0.15	−0.03
12	1	0.18	0.05	−0.00
13	0	0.03	−0.06	−0.03

*ΔFMA, Difference between FMA scores following stroke and following intervention; ΔGC, Difference between GC-values following stroke and following intervention*.

## Discussion

In this study, we used a spectral GC approach on resting-state fMRI data of 30 participants to investigate the organization of motor-execution network for young able-bodied and aged stroke survivors along with substantial changes after the stroke survivors underwent mental practice alone or combined mental and physical therapy. We found that node and network activities were dominant in the frequency band 0.06–0.08 Hz for all the ROIs in all conditions. As expected we found that node activity for each ROI was significantly higher in AB condition than SS condition but unexpectedly there was no significant difference between node activities for SS, MP, and MP+PT conditions. There were bidirectional causal influences between LM1 and SMA, RPMC and SMA, RPMC and LM1, SMA and RM1, and SMA to LPMC for young able-bodied participants, but none of the directions were significant for stroke survivors even when they underwent a session of MP. Some of the connections, for example between LM1 and SMA and from SMA to LPMC, showed significant causal flow when stroke survivors underwent combined session of MP and PT (MP+PT). Percent decrease in connection strength reflected by causal flow for aged stroke survivors compared to young able-bodied ranged from −21 to −97% whereas the percent modulation for stroke survivors with MP and MP+PT compared to those individuals receiving no treatment ranged between 18–65 and 45–94%, respectively. Furthermore, as predicted young able-bodied participants demonstrated significantly stronger network causal flow than aged stroke survivors. There was no significant difference between network causal flow before and after the MP treatment in stroke survivors. But, to our surprise the causal flow was significantly more after MP+PT than before any treatment. Furthermore, network causal flow after MP+PT was also found to be significantly more than after MP only. We also found that the FMA scores were significantly higher following intervention (MP+PT) in post-stroke hemiplegic patients indicating a greater degree of recovered upper limb function in this group. There was a correlation, which tended toward significant value, between difference in FMA scores and difference in directed functional connectivity measures from SMA to LPMC following stroke and when the stroke-survivors underwent MP+PT.

### Low-frequency network activity

Intrinsic functional networks usually show coherent oscillatory activity in the low frequency band, less than 0.1 Hz. Spontaneous synaptic activity of neurons is known to give rise to fluctuations in fMRI BOLD signals. These low-frequency oscillations are believed to mediate long-distance synchronization of distributed brain regions, modulation of which represent cortical excitability (Buzsáki and Draguhn, 2004; Bajaj et al., 2013, 2014). Further evidence points to the notion that these oscillations have a definite neuronal basis rather than the result of physiological artifact (Lowe et al., 1998; Cordes et al., 2001; Bajaj et al., 2013). The resting-state activity and the spontaneous fluctuations also reflect the dynamic self-organizing nature of brain (Raichle and Mintun, 2006). The power of such low-frequency fluctuations of brain signals may differ significantly between stroke survivors and able-bodied healthy individuals (Tuladhar et al., 2013), which is consistent with our results. Our findings are consistent with a study by Tsai et al. (2014) who reported that during the resting-state, the amplitude of low frequency oscillations is altered in people with impaired consciousness following a stroke. Significant differences in the amplitude of low frequency oscillations was also reported during resting-state in the brain areas of people suffering from depression (Wang et al., 2012).

However, it has been postulated that following a stroke, brain network activity may deviate. Fluctuations with frequency less than 0.1 Hz have been shown to contribute to resting-state functional connectivity in auditory, visual and motor cortices (Cordes et al., 2001). Strong coherence relationship between motor areas have been found in the frequency band 0.02–0.15 Hz during rest as well as in the presence of lesions (Otten et al., 2012). Dominance of ultra-low frequency band (0.01–0.06 Hz) in cortical networks and of 0.01–0.14 Hz in limbic networks suggest the involvement of distinct frequency bands in the resting-state fMRI signals (Wu et al., 2008).

### Altered functional connectivity following stroke

Detailed descriptions of resting-state connectivity in stroke survivors may help rehabilitation scientists recognize and target insulted neural networks with evidence-based therapies. It has been suggested that coupling between distinctive cortical areas and their functionality following stroke can be better understood in the absence of any active task (Grefkes and Fink, 2011). The degree of network disturbance and reduction in network activity following stroke is mainly caused by weak or abnormal neural coupling between higher order pre-motor and motor areas and is dependent on the age, location of lesion and intensity of anatomical damage (Grefkes and Fink, 2011; Sun et al., 2012). Stroke may also leave a strong negative impact on the coupling between the cortex and spinal cord and among cortical areas, which are contiguous or removed from the location of lesion (Grefkes and Fink, 2011). Our findings are consistent with a dynamic causal modeling (DCM) study by Rehme and colleagues, where changes in effective connectivity within M1, PMC, and SMA were observed following stroke (Rehme et al., 2011) i.e., there was reduction in positive coupling of SMA and PMC with M1. In another DCM study of 12 subacute stroke patients during a hand movement task, Grefkes and colleagues found intrahemispheric and interhemispheric disturbances due to subcortical lesions (Grefkes et al., 2008b). They reported that the intrinsic neural coupling between SMA and M1 was significantly reduced in patients recovering from stroke. The deficiency in motor skills due to a single subcortical lesion was thought to be related to pathological interhemispheric interactions among core motor regions. In comparison to able-bodied participants, weaker paths weights have been found from PMC to M1 for stroke patients (Inman et al., 2012). Patients with stroke had significantly diminished connections between fronto-parietal cortices and primary motor areas, suggesting an overall weaker confirmatory model. Our findings also showed a significantly diminished motor network compared to young healthy participants. In addition, abnormal effective connectivity has been shown between PMC, SMA and prefrontal cortex in patients with Parkinson's disease due to disturbed functionality of a subcortical circuit (Rowe et al., 2002).

### Recovered functional connectivity following rehabilitation

Several studies on animals and humans provide insight demonstrating the basis of recovery mechanisms. Studies in rodent models have shown multiple cellular level changes occur in the unaffected hemisphere during recovery from stroke (Jones and Schallert, 1994). A study on non-human primates have shown that the degree of motor impairment after stroke depends upon the damage to direct corticospinal connections between neurons in motor areas M1, PMC, and SMA and alpha-motor neurons (Dum and Strick, 2002; Grefkes and Fink, 2011). Motor recovery may be associated with increased activation in the SMA (Aizawa et al., 1991). Various hypotheses have been proposed describing the source of activations in SMA. It is believed that without execution of a motor plan, MP or mental rehearsal forms a hypothetical environment of movements, which causes activation of motor preparation or motor execution network (Jeannerod and Frak, 1999). Lotze et al. (1999) in an fMRI study of healthy participants have verified this observation, where supplementary motor area (SMA), premotor cortex (PMC), and primary motor area (M1) are found to be consistently active during motor execution as well as during motor imagery task. Activation of the same neural populations during MP and physical actions may be because of the same vegetative responses elicited by both (Butler and Page, 2006). Performance times are also found to be close for imagined and physically performed tasks with different levels of difficulty (Kohl and Fisicaro, 1995; Cerritelli et al., 2000). Treatment by MP, which is fundamentally rehearsal of an action mentally without any physical effort, is usually considered as a mental imagery (MI) task. Only slight but insignificant restoration of insulted brain networks following MP has been observed in the current study which may be because both motor-imagery and motor-execution are known to associate with similar brain networks. Brain studies have confirmed a correspondence between imagined and executed movements and considered MI as a dynamic process with a strong correlation with motor-execution. Mental rehearsal by itself or in combination with physical practice has been proven to be beneficial for healthy as well as for mentally challenged individuals (Sharma et al., 2006; Dickstein and Deutsch, 2007). Our report that MP with motor imagery may cause the internal simulation of movements but not of a sufficient intensity to match that of able-bodied participants. Whereas repetitive physical practice combined with MP causes a stronger cortical reorganization with concomitant improvement in function is consistent with previous findings (Jackson et al., 2003; Butler and Page, 2006). For comparison, previous neuroimaging studies suggest that during resting state, there is significant influence of age on functional connectivity within the motor network and normal aging may cause disruption and decline of function in motor areas (Wu et al., 2007a,b; Solesio-Jofre et al., 2014). This may explain why young able-bodied participants demonstrated significantly stronger network causal flow than aged stroke survivors; in all the conditions, especially before any treatment. But on the other hand, in a resting state fMRI study on stroke survivors, Carter and colleagues confirmed that lesions are responsible for changes in the functional architecture of the brain as well as constrain behavioral outputs (Carter et al., 2010).

Furthermore, our findings of the directed functional connectivity changes for stroke patients following rehabilitation are consistent with a study by Rehme and colleagues who reported an increase in coupling between SMA, M1, and PMC following rehabilitation (Rehme et al., 2011). SMA and PMC are found to have direct extensive projections to M1 in non-human primates (Dancause et al., 2005) and may play a critical role in motor recovery. Findings from a study by James and colleagues suggested that the unaffected hemisphere has a strong and direct influence on the affected hemisphere following stroke, but this influence diminishes with recovery (James et al., 2009). Despite the variability due to heterogeneity of lesion locations in our sample of stroke-survivors, our current findings suggest a significant influence of rehabilitation therapy (i.e., MP+PT) on motor networks and upper limb motor recovery in post-stroke hemiplegic patients.

Previous studies (Page et al., 2001; Butler and Page, 2006; Confalonieri et al., 2012) have shown that the combination of MP and PT is helpful in improving functional and motor skills more than MP only. MP by itself is considered an effective technique to enhance motor performance by tracing the overlap between motor imagery and motor execution neural circuits (Jeannerod, 2006). Although, the improvement in muscular strength of participants with deficiency in motor skills following MP is less than physically trained participants (Yue and Cole, 1992). We found that the combination of MP and PT significantly improved the connectivity between specific cortical areas as well as for motor-execution network as a whole and tended toward connectivity values of healthy participants. These findings are in-line with our behavioral results where we reported that the FMA scores for patients who received MP+PT are significantly higher than before intervention. Differences in FMA scores and GC values before and after MP+PT also follow a linear trend. Page and colleagues also observed that the patients who received MP+PT improved significantly by an average of 7.81 and 6.72 points on the Action Research Arm (ARA) test and Upper Extremity Fugl–Meyer Assessment of Motor Recovery After Stroke (FM) respectively whereas patients who received PT and relaxation showed significantly lower scores of only 0.44 points and 1 point on the ARA and FM, respectively (Page et al., 2007).

We also found that there was decrease in causal flow values from SMA to RM1, RPMC to LM1, and RPMC to SMA after MP. The decrease in causal values was less when stroke patients underwent MP+PT. The decrease in value could be because mental practice or imagery usually consists of a set of relatively independent processing sub-systems (Kosslyn et al., 1984, 1990). Lack of simultaneous activations in these sub-systems may result in weakening of the connections in motor network. Mental practice may also involve some manipulation, producing descriptions of the task or daydreaming (Kosslyn et al., 1990; Schuster et al., 2012). Hence, whether and how long these weak interactions arising from mental practice are retained is an interesting question for future investigations.

### Limitations

Lesion locations in our sample of participants were not homogeneous. This may have added variability to the connectivity measures for some of the regions of interest. The sample included stroke survivors with a wide age range and time since stroke, hence further adding to intersubject variability. Future studies having participants with age-matched stroke and able-bodied volunteers can provide better references for brain connectivity comparisons and may give better estimates of connectivity improvements compared with able-bodied patients. Despite the variability and this limitation, our data show excellent correlation between brain network activity flow and behavioral measures within the recovering stroke patients of similar age group.

In conclusion, the results of the current study suggest that the fMRI BOLD brain signals can capture the network activity flow changes within the cortical motor-execution networks following stroke and during the course of rehabilitation and recovery. The combination of mental practice and physical therapy is an effective treatment option, capable of producing significant behavioral and brain activity changes. The directed functional connectivity approach allows us to probe the brain network mechanisms during the course of motor recovery from stroke, providing the basis for clinical decisions making and selection of treatments for stroke patients.

### Conflict of interest statement

The authors declare that the research was conducted in the absence of any commercial or financial relationships that could be construed as a potential conflict of interest.

## Abbreviations

SS: stroke survivors
MP: mental practice
PT: physical therapy
MP+PT: combination of mental practice and physical therapy.
